# Capillary Dilation and Rarefaction Are Correlated with Intracapillary Inflammation in Antibody-Mediated Rejection

**DOI:** 10.1155/2014/582902

**Published:** 2014-02-10

**Authors:** Xue Li, Qiquan Sun, Mingchao Zhang, Kenan Xie, Jinsong Chen, Zhihong Liu

**Affiliations:** ^1^National Clinical Research Center of Kidney Diseases, Jinling Hospital, Nanjing University Clinical School of Medicine, 305 East Zhong Shan Road, Nanjing 210002, China; ^2^Department of Renal Transplantation, The Third Affiliated Hospital, Sun Yat-Sen University, Guangzhou 510760, China

## Abstract

Antibody-mediated rejection (ABMR) remains one of the major causes of graft loss after renal transplantation. It is dominated by endothelial damage in microcirculation. Clarifying the mechanism of microcirculating damage is obviously a key step to understand the pathogenesis of ABMR. Here we characterized capillary variation in ABMR and its possible mechanisms. Compared with T cell-mediated rejection and stable grafts, there was a significant dilation and rarefaction in peritubular capillaries (PTCs) of the ABMR group; Image-Pro Plus revealed a significantly larger intra-PTC area. Interestingly, the dilation of PTCs was strongly correlated with the intra-PTC cell counting. Moreover, peritubular capillary inflammation is correlated with *in situ* T-bet expression, and there was a good correlation between the intra-PTC expression of T-bet and the PTC diameter. HIF-1**α** up-regulation could be observed in ABMR but it was not necessary for capillary dilation. In general, ABMR is characterized with early capillary dilation and rarefaction; our data confirmed that the dilation is strongly correlated with intracapillary inflammation, which in turn is correlated with *in situ* T-bet expression. T-bet plays an important role in the development of microcirculating injury, and thus it is a potential target for the treatment of ABMR.

## 1. Introduction

Antibody-mediated rejection (ABMR) is a recalcitrant entity with great impact on patient and graft survival [1, 2]. In the past decade, improvements in HLA technology along with the recognition of the role of C4d in ABMR have revolutionized the understanding of this important entity, and significant advances have occurred in the treatment of ABMR [3–5]. However, the mechanism of ABMR is far from being fully elucidated, and the long-term survival of these allografts is greatly reduced when compared to that of grafts without rejection or history of T cell-mediated rejection (TCMR) [2, 6].

ABMR is dominated by endothelial damage in microcirculation [7, 8]. Microcirculation inflammation, including glomerulitis and peritubular capillaritis (PTCitis), has been recognized as a cardinal feature in the diagnosis of ABMR [9, 10]. Peritubular capillary dilation is another important side of microcirculation changes, and although it has been noticed in the ABMR for years [2], it is far from being clearly demonstrated and its pathogenesis remains unclear. The assessment of capillary dilation will be helpful to clarify the mechanism of ABMR.

T-bet is a member of the T-box family of transcription factors regulating Th1 lineage commitment [11]. In a recent study, we found that transplant glomerulopathy, a principal form of late ABMR, had a significant increase in T-bet expression in peritubular capillaries (PTC), and this expression was strongly correlated with the count of intra-PTC inflammation cells. Furthermore, PTC dilation was also strongly correlated with the intra-PTC inflammation [12]. In a previous study, we found intraglomerular inflammation correlated with *in situ* expression of T-bet in patients with ABMR [13]. We hypothesize that T-bet expression might be also correlated with PTC injury in early ABMR.

HIF-1 is a transcription factor which acts as a master regulator coordinating oxygen homeostasis [14], and the HIF system is ubiquitous which is instantaneously up-regulated upon hypoxia [15]. In ABMR, whether PTC injury can cause tissue damage via hypoxia is unknown. In this study, we explored the dilation of PTC in relation to inflammation, T-bet expression, and hypoxia. Our data provide novel insight into the development of antibody-mediated graft injury.

## 2. Materials and Methods

### 2.1. Patient Selection

The patients were retrospectively selected from among 226 renal allograft recipients who had performed renal biopsy between June 2008 and May 2012 at Jinling Hospital, Nanjing University School of Medicine, Nanjing, China. Among them, 18 recipients were diagnosed as having C4d-positive acute rejection episodes according to clinical manifestations and histological features. The diagnosis was based on the following: (1) clinical evidence of acute rejection, manifested as rapid renal dysfunction and/or decrease of urine volume; (2) C4d deposition in the PTC area; and (3) pathologic features that met Banff's 1997 criteria for acute rejection grade I, II, or III. Additionally, rejection episodes occurring beyond the first month after transplantation are more likely to offer a mixed histologic picture, often demonstrating acute and chronic, vascular and tubulointerstitial, pathology. Thus, we examined only biopsies from recipients in the first posttransplant month. This group was compared with a group of TCMR patients who were diagnosed within the same time period. Since PTCitis (ptc) and glomerulitis (g) are often associated with ABMR and g + ptc = 0 was confirmed to be a useful diagnostic algorithm for TCMR exclusion [16], we excluded all the recipients with PTCitis and glomerulitis in TCMR group. These patients were randomly matched with a group of recipients with stable graft function who received protocol biopsies as controls. In addition, we also included a TG group to compare PTC variation with ABMR. We applied the following inclusion criteria: (1) biopsy confirmation of the presence of a duplication of the glomerular basement membrane on periodic acid-Schiff or silver stain, and (2) 1 year of follow-up after the diagnosis. An electron microscopic evaluation was performed to exclude membrane duplication that was caused by recurrent or de novo glomerular disease. Informed consent was obtained from all patients, and the Human Subjects Committee of Jinling Hospital, Nanjing University School of Medicine approved all of the study protocols.

### 2.2. Renal Biopsies

Protocol biopsies were performed between days 12 and 17 posttransplantation (so-called 2-week protocol biopsy), and diagnostic biopsies were performed upon clinical indication and according to local standard of practice. All rejection episodes were proved by biopsy. Two needle biopsy cores were obtained from each renal allograft for morphologic study: one for formalin fixation and the other for quick-freezing. Hematoxylin and eosin, periodic acid Schiff, methenamine-silver, and Masson stains were routinely used on the formalin-fixed tissue. The residual biopsy tissues were stored for future use. Fresh-frozen tissues were analyzed by immunofluorescence microscopy using a conventional panel of antibodies against IgG, IgM, IgA, C3, C4, C1q, and C4d. C4d staining was routinely performed on frozen slides using an indirect immunofluorescence technique with a primary affinity-purified monoclonal antibody (mouse anti-human; Quidel, San Diego, CA, USA) and an FITC-labeled affinity-purified secondary rabbit anti-mouse IgG antibody (Dako, Denmark). The staining was performed using standard procedures. Positive C4d staining was defined as a bright linear stain along the capillary basement membranes that involved over half of the sampled capillaries in accordance with the 2001 Banff Meeting [17].

### 2.3. Immunohistological Analysis

CD4, CD8, CD68, and CD20 were regularly detected when a biopsy was performed. The intragraft expression of HIF-1*α* and CD31 was retrospectively studied via immunohistochemistry using stored residual biopsy tissues. Immunohistochemistry was performed on formalin-fixed, paraffin-embedded tissues. The antibody regimens were conducted as follows: mouse monoclonal antibody HIF-1*α* (Novus Biologicals, Littleton, CO, USA); mouse monoclonal antibody against T-bet (Santa Cruz Biotechnology, Santa Cruz, CA); and mouse monoclonal antibodies against CD4 (Novocastra, Newcastle upon Tyne, UK), CD8 (Novocastra), CD68 (Dako, Carpinteria, CA, USA), CD20 (Dako), and CD31 (Dako). The sections were reviewed by two pathologists, and the results are expressed as the total number of positive cells per glomerulus or per square millimeter in the cortex.

### 2.4. Microvascular and Intracapillary Cell Counting

To assess the number and size of PTCs, a CD31 costaining was used as previously described [18, 19]. The PTC density was calculated by counting the total number of PTCs within the confines of each of 10 random 0.25 mm^2^ fields (each of these fields was delineated by a 1 cm^2^ ocular grid that was viewed at ×400 magnification), and the result is expressed as the mean per field. The diameter of these PTCs was also measured, and their mean was calculated in micrometers. To measure the PTC spaces, slides from each case were examined using an Olympus IX70 inverted system microscope (Olympus America, Melville, NY, USA) connected to a Hewlett Packard computer with Image-Pro Plus software (Media Cybernetics, Silver Springs, MD, USA) [20]. The intra-PTC area is expressed as the proportion of total PTC spaces over the entire cortex field. Intraglomerular capillaries were measured for their quantity (capillary counts per glomerulus) and diameter on cross-section of the glomeruli. To calculate the intracapillary inflammation cells, costaining of CD31 and inflammation markers (e.g., CD4, CD8, and CD68) were performed, and the results are expressed as the mean per PTC or glomerulus. Glomerular cross-sectional area was also determined using the Image-Pro Plus software.

### 2.5. Statistical Methods

Statistical analyses were conducted using SPSS (v16.0) and GraphPad Prism (v5) software. Pairwise comparisons of variables based on proportions were done by Fisher's exact test with Bonferroni correction for *P* value. Continuous variables were presented as mean ± s.d. and compared using one-way analysis of variance (ANOVA) followed by post hoc pairwise comparisons using LSD tests, or analyzed using nonparametric method if the data were not normally distributed. Ordered categorical data were presented as median (25th–75th percentiles) and compared using the nonparametric Kruskal-Wallis ANOVA on ranks for global comparison, followed by Duncan's analysis for multiple comparisons. Spearman's correlation was used in analysis correlation. The level of statistical significance was set at *P* ≤ 0.05 (two-sided).

## 3. Results

### 3.1. Baseline Patient Characteristics

Forty-five renal allograft recipients were included in this study, including 18 cases of ABMR, 13 cases of TCMR, and 14 cases of stable grafts as controls. The diagnosis of ABMR and TCMR was based on Banff 05 [21]. The baseline patient characteristics are listed in Table 1. None of the recipients had previously received an organ transplant. There were no significant differences among the three groups with respect to patient age, gender, time of prior transplantation, time of biopsy, or incidence of positive panel-reactive antibody. Each patient received anti-IL-2 receptor monoclonal antibody for the induction of immunosuppressive therapy and was subsequently maintained on a similar immunosuppressive protocol after transplantation (Table 1).

### 3.2. Pathology Findings

A comparison of histological lesions in the three groups is given in Tables 2 and 3. Recipients with lymphocytes infiltration in PTCs or glomeruli were excluded from TCMR group while PTCitis was observed almost in all patients in ABMR group. Tubulitis was seen in all patients of acute rejection enrolled in the study, compared with 42.9% of stable graft. Significantly more patients (88.9%) have intimal arteritis in the ABMR group compared with either of the other two groups. We used immunohistochemistry to detect CD4, CD8, CD68, and HLA-DR expression. In the ABMR group, the average values for CD4, CD8, CD4/CD8 and CD68, were all similar to the TCMR group. However, when compared with stable grafts group, every kind of lymphocyte is significantly higher in both ABMR and TCMR groups.

### 3.3. ABMR Is Associated with Peritubular Capillary Dilation and Rarefaction

**Figure 1:**
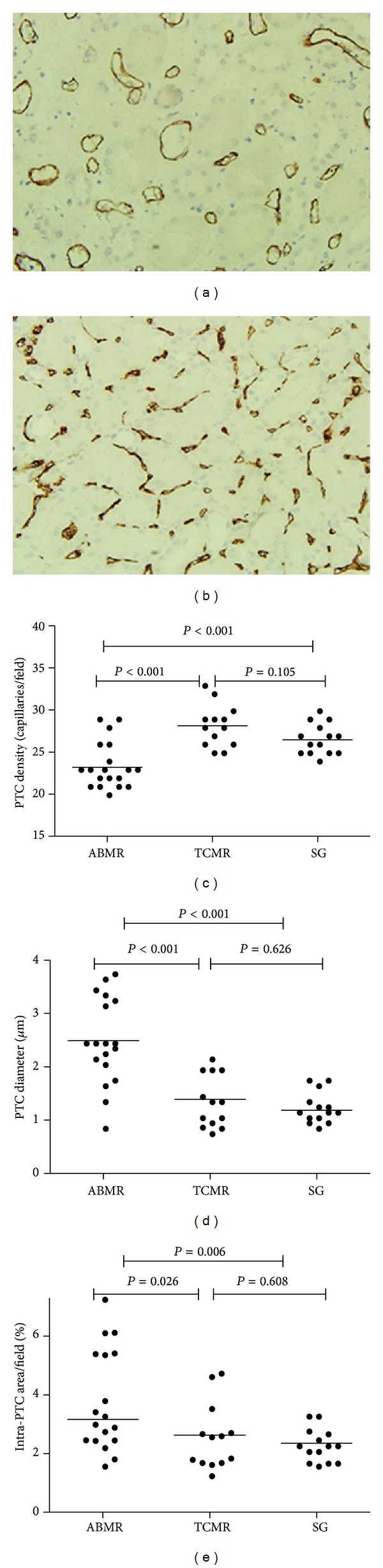
Peritubular capillary (PTC) dilation and rarefaction in antibody-mediated rejection (ABMR). Labeling the endothelial cells for CD31 showed PTC dilation and rarefaction in ABMR group (a), compared with PTC staining in T cell-mediated rejection (TCMR) group (b). (c) PTC density among the three groups revealed a rarefaction of PTCs in ABMR group. (d) PTC diameters among the three groups revealed significant PTC dilation in ABMR group. (e) Intra-PTC areas among the three groups revealed significant PTC enlargement in ABMR group. (a, b) Original magnification, ×400.

Labeling the endothelial cells for CD31 made it possible to calculate the number and diameters of the capillaries. Figures 1(a) and 1(b) show that CD31 staining labeled the capillaries notably well. Overall, ABMR was correlated with an increased PTC diameter and a decreased PTC density.

We compared the PTC density and capillary diameter among the three groups (Figures 1(c) and 1(d), Table 4). In the ABMR group, the density of PTCs was significantly lower compared with the stable graft group (22.22 ± 2.51 versus 25.64 ± 1.82/field, resp., *P* < 0.001), whereas the diameter of the existing PTCs was significantly larger (2.34 ± 0.81 versus 1.09 ± 0.29 *μ*m, resp., *P* < 0.001), suggesting a rarefaction and dilation of the capillaries (Figure 1(a)). However, in the TCMR group, we found no such rarefaction and dilation of PTCs as observed in the ABMR group (Figure 1(b)). The capillary density was similar between the TCMR group and protocol biopsies (27.23 ± 2.49 versus 25.64 ± 1.82/field, resp., *P* = 0.105). In addition, Image-Pro Plus revealed a significantly larger intra-PTC area in the ABMR group (0.035 ± 0.018) compared with the TCMR (0.023 ± 0.012, *P* = 0.026) and stable graft (0.020 ± 0.006, *P* = 0.006) groups (Figure 1(e)). In addition to PTCs, capillary dilation was also observed within the glomeruli. However, there were no significant differences between the three groups in the diameter of the intraglomerular capillaries and intraglomerular capillaries' area.

### 3.4. PTC Dilation Is Correlated with Microcirculation Inflammation

**Figure 2:**
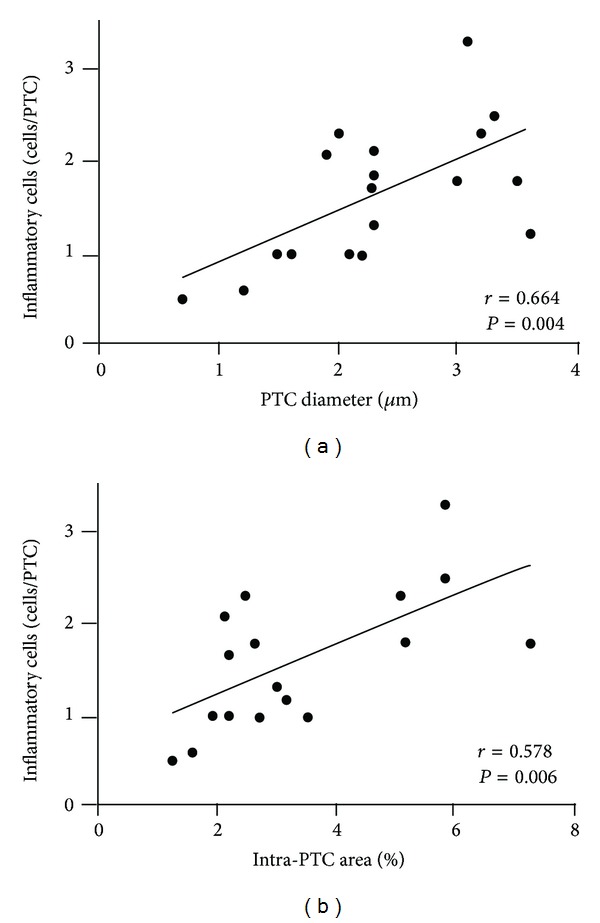
The relationship between microcirculation inflammation and peritubular capillary (PTC) dilation. In the antibody-mediated rejection group, PTC diameter (a) and intra-PTC area (b) were strongly correlated with the number of intra-PTC cells.

In the ABMR group, we detected a significant correlation between PTC dilation and microcirculation inflammation. The dilation of PTCs was strongly correlated with the quantity of intra-PTC infiltrating cells (*r* = 0.664, *P* = 0.004), as shown in Figure 2(a). In contrast, as shown in TCMR group (Figure 1(b)) and SG group (not shown), capillary dilation was rare, and it was also found to mostly coexist with luminal inflammatory cells. We also calculated the ratio of the total intra-PTC area within the cortex to the area of the entire cortex in ABMR group, and found that this ratio was also strongly correlated with intra-PTC cell counting (*r* = 0.578, *P* = 0.006) (Figure 2(b)).

### 3.5. Microcirculation Inflammation Is Correlated with *In Situ* T-bet Expression

**Figure 3:**
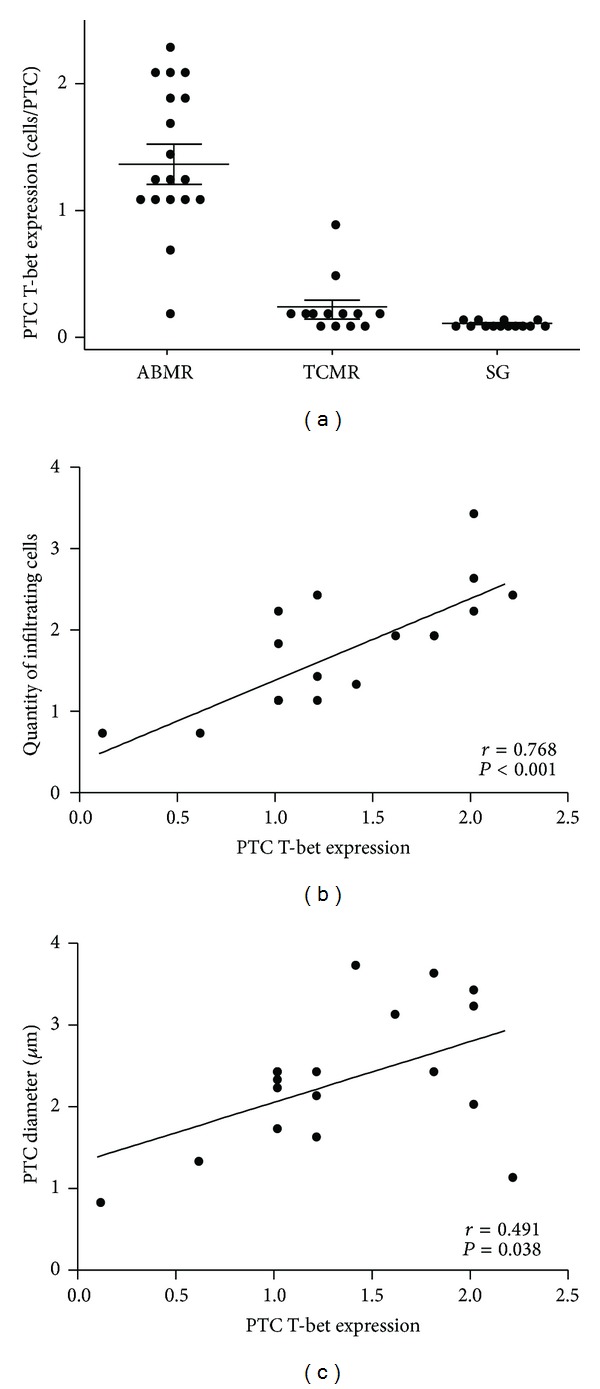
T-bet expression in antibody-mediated rejection (ABMR). (a) Quantitative measurement of the number of intra-PTC T-bet-expressing cells in the ABMR, T cell-mediated rejection (TCMR), and stable graft (SG) groups. In the ABMR group, T-bet expression was strongly correlated with the quantity of infiltrating cells (b) and peritubular capillary (PTC) diameter (c).

A significant increase in T-bet expression was detected in the PTCs, and the majority of T-bet+ cells were typically located within the capillary lumen. Intra-PTC T-bet expression was also detected in the TCMR group; however, its expression was much lower than that in the ABMR group (1.34 ± 0.56 versus 0.15 ± 0.22/capillary, *P* < 0.001) (Figure 3(a)). In the ABMR group, the T-bet expression correlated well with the quantity of infiltrating cells (*r* = 0.768, *P* < 0.001) (Figure 3(b)). Moreover, since PTC dilation is correlated with microcirculation inflammation as above mentioned, we also find a good correlation between the intra-PTC expression of T-bet and the PTC diameter (*r* = 0.491, *P* = 0.038) (Figure 3(c)).

### 3.6. HIF-1*α* Expression Is Correlated with PTC Dilation in Both ABMR and TCMR

**Figure 4:**
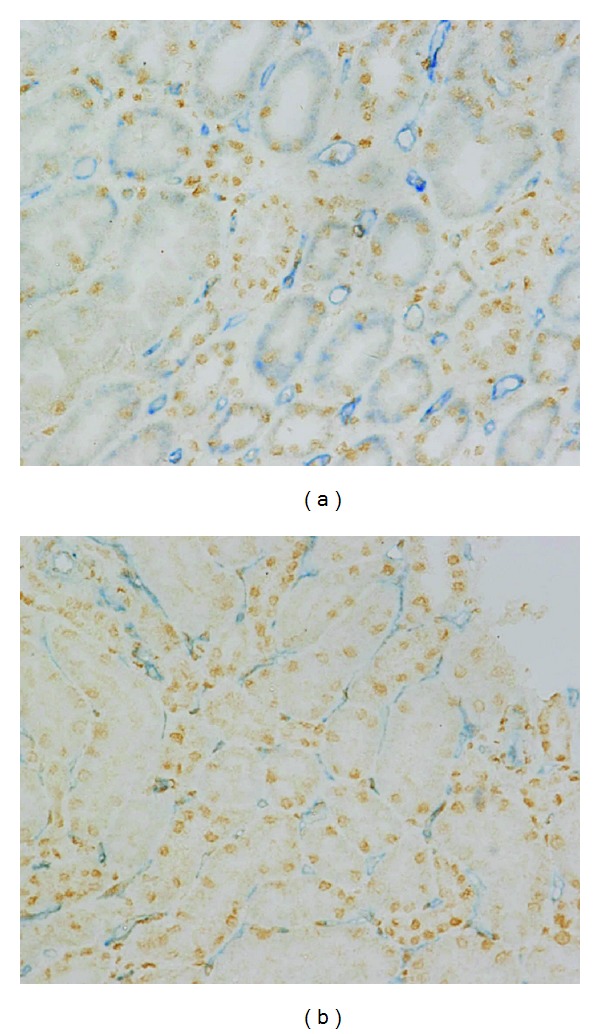
HIF-1*α* expression in antibody-mediated rejection (ABMR) and T cell-mediated rejection (TCMR). Double staining of CD31 and HIF-1*α* showed capillary dilation, and HIF-1*α* expression occurred somewhat in parallel both in ABMR (a) and TCMR (b) while HIF-1*α* expression was not necessary for capillary dilation. Original magnification, ×400.

Similar to the findings in experimental acute renal failure [22], the strongest and most abundant signals were detected in the medulla, and signal intensity and abundance increased from the outer medulla to the deep papilla. Moreover, cortical tubular immunostaining for HIF-1*α* was positive and even signals can be detected in some glomeruli. According to the results of double staining of CD31 and HIF-1*α*, capillary dilation and HIF-1*α* expression occurred somewhat in parallel both in ABMR and TCMR. It is noteworthy that, as shown in Figure 4, PTC dilation could be observed where HIF-1*α* was up-regulated while HIF-1*α* expression was not necessary for capillary dilation.

### 3.7. Capillary Variation between ABMR and TG

**Figure 5:**
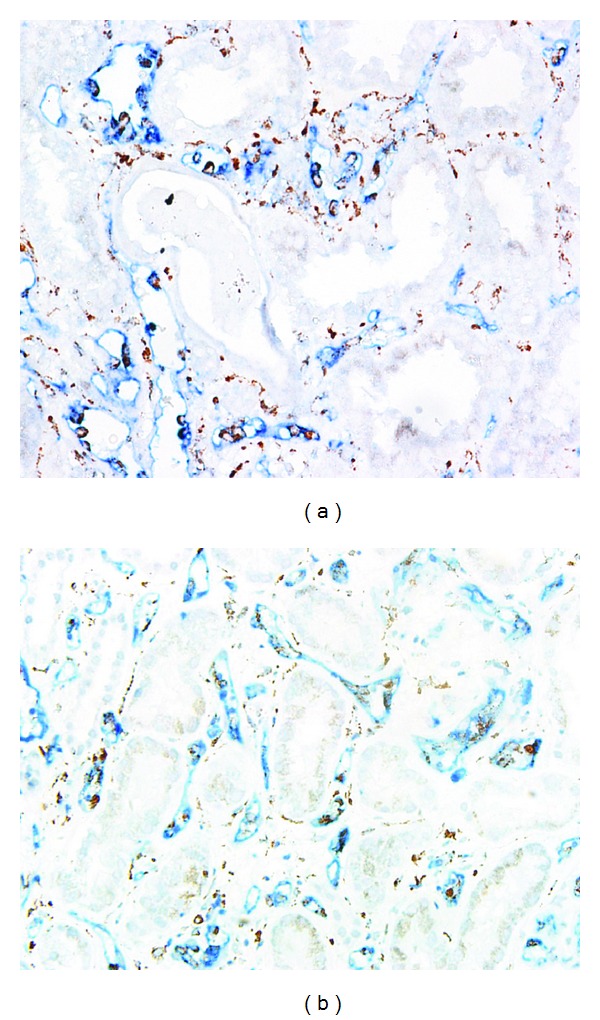
Capillary variation between antibody-mediated rejection (ABMR) and transplant glomerulopathy (TG). CD31 and CD68 (labeled in brown) costaining showed peritubular capillary (PTC) dilation was much more severe in the TG group (a) than in the ABMR group (b).

In order to compare the capillary variation between ABMR and TG, 32 cases of TG were selected as control group (the baseline characteristics previously described in [12]). We found that the PTC loss is similar in two groups, while the PTC diameters were significantly larger in TG group (2.34 ± 0.81 versus 6.35 ± 2.16, *P* < 0.001), leading to much more severe PTC dilation. Moreover, the diameters of capillary loops within glomerulus were also increased, and the intraglomerulus area was also significantly increased and showed an enlarged glomerulus. Anyway, the sizes of glomerulus as well as capillary loops within glomerulus were similar between ABMR and TCMR cases. (Figure 5 and Table 5).

## 4. Discussion

This study revealed that ABMR is associated with peritubular capillary dilation and rarefaction. We confirmed that PTC dilation is correlated with microcirculation inflammation, and PTC inflammation is in turn strongly correlated with* in situ* T-bet expression. These data suggest that T-bet plays an important role in the pathogenesis of microcirculation injury in ABMR. These findings shed a new light on the pathogenesis of ABMR.

Though it is the general belief that ABMR is associated with peritubular capillary dilation [23–25], it is not until this study that dilation has been demonstrated clearly in this setting. Our study clearly demonstrates that the PTC diameter and intra-PTC area were significantly increased in the ABMR. Shortly after the development of ABMR, the diameters of PTCs are increased to twice of the control group, which leads to significantly increased intra-PTC area. Nevertheless, the dilation is diffused and can be seen in most of the PTCs, no matter in cortex or medulla. This variation can be regarded as a feature of ABMR as it cannot be observed in TCMR. As the diameter of PTC is easy to measure and evaluate throughout the whole specimen, it may be applicable to biopsy interpretation and may be of good diagnostic utility for ABMR.

Moreover, our data also revealed a rarefaction of PTC during ABMR. To our knowledge, this is the first report on PTC loss when ABMR occurs. Although the diameter of each PTC is increased, the count of PTC is significantly decreased (Figure 1, Table 4) which can be observed in early biopsies immediately after the ABMR occurs. The quick rarefaction of PTC seems to be unique in early ABMR as it is not observed in TCMR or TG. PTC loss has been demonstrated in models involving ageing, cyclosporine, angiotensin II infusion, chronic catecholamine infusion, glomerulonephritis, radiation-induced injury, and potassium depletion [26], which mostly are involved in a local alteration in the balance between angiogenic and angiostatic factors [27, 28]. However, the quick PTC rarefaction during the rejection suggests a dramatic process, which is most probably caused by direct damage to the capillary wall. PTC loss after renal transplantation usually associates with increased interstitial fibrosis/tubular atrophy, and predicts reduced renal function [19]; thus the dramatic PTC loss during ABMR is an important process to graft dysfunction.

The pathogenesis of PTC variation during ABMR is not clear; however, as the dilation can be observed quickly when ABMR occurs, it should be a response to the antibody activity. Our data revealed that the PTC dilation is strongly correlated with intra-PTC inflammation. Although interstitial inflammation might cause quick vessel dilations in the early phase [29], the PTC dilation is more likely to be related to intra-PTC inflammation, as the dilation cannot be observed in TCMR cases, which have more severe interstitial inflammation. Moreover, the dilation of the PTC lumen was strongly correlated with the degree of microcirculation inflammation, whereas PTC dilation was mostly observed in areas with PTC inflammation. This finding demonstrates a strong correlation between capillary dilation and PTC cellular infiltration. This phenomenon can be seen in TG as well and it is quite likely that the capillary dilation is caused by *in situ* inflammation. Those data suggest a central role of inflammation in the development of microcirculating variation during ABMR.

Furthermore, we find that intra-PTC inflammation is strongly correlated with *in situ* expression of T-bet. This correlation is unique to the ABMR group, as the TCMR and stable graft groups, which rarely have microcirculation inflammation, only rarely exhibited T-bet expression in the PTC areas. T-bet is a member of the T-box family of transcription factors and is a key determinant of T-helper cell differentiation into Th1. Our previous study showed that T-bet expression is correlated with glomerulitis in ABMR, further study revealed that both glomerulitis and PTCitis are correlated with T-bet expression in TG, which is a chronic form of ABMR. This study proved that both kinds of microcirculating inflammation, PTCitis and glomerulitis, are correlated with T-bet expression, no matter in acute ABMR or chronic type. Higher T-bet expression is correlated with more PTC inflammation, and in turn is correlated with larger PTC diameters. This study suggests that T-bet pathway might be a potential target in the management of ABMR.

As capillary damage may lead to local hypoxia, it is possible that hypoxia is involved in the pathogenesis of ABMR. At experimental level, there has also been described an intense dilation of the peritubular capillaries in chronic allograft dysfunction [26, 30], and the morphological changes were thought to be related to chronic ischemia [26]. We hypothesized that hypoxia takes part in the development of PTC dilation. Since HIF allows for hypoxia detection at a single cell resolution [31–33], and as previously described in vivo [34], renal HIF-1*α* immunostaining was almost exclusively found in tubular segments, HIF-1*α* immunostaining had been performed in this cohort. Somewhat surprising, PTC dilation could be observed where HIF-1*α* was up-regulated while some dilation had nothing to do with HIF-1*α* expression, which suggests that hypoxia is not necessary for PTC dilation in ABMR. Obviously the PTC dilation in ABMR has a pathway that is different to chronic allograft dysfunction.

Current data also showed that the microcirculating variation in ABMR is similar to its chronic pattern, TG. Both ABMR and TG have significant PTC dilation, which is correlated with PTC inflammation, and in turn with *in situ* T-bet expression. It suggests that both acute and chronic patterns of humeral rejection share a common mechanism of PTC variation; T-bet expression might account for the development of microcirculation inflammation. However, in TG, the PTC dilation is much more severe than in ABMR, and there are enlarged glomerulus loops and glomerulus size, which are not shown in the ABMR group. Those changes might be caused by continuous antibody activity.

The diagnosis of ABMR has largely depended on the C4d staining in the past decade. However, in spite of high specificity, C4d is lacking sensitivity, and many cases of rejection with anti-HLA are C4d negative [7, 35–37]. The variation of PTC during ABMR, including PTC dilation and rarefaction, differs to TCMR and may be used in the diagnosis of ABMR. Moreover, our previous study had reported that the predominance of T-bet over GATA-3 may distinct ABMR from TCMR [13]; the current data even showed that the predominance of T-bet expression in PTC is a feature of ABMR and can also be regarded as a diagnosis marker.

In summary, this study shows that ABMR is associated with PTC dilation and the latter is correlated with microcirculation inflammation, and microcirculation inflammation is strongly correlated with *in situ* T-bet expression. These results suggest that inflammation may take part in the pathogenesis of PTC dilation, and they warrant further investigation.

## Figures and Tables

**Table 1:** Clinical characteristics of patients who participated in this study.

Characteristics	ABMR (*n* = 18)	TCMR (*n* = 13)	SG (*n* = 14)	*P* value
Gender, male (%)	9 (50.00)	10 (76.92)	10 (71.42)	0.244
Age (years)	40.72 ± 7.09	39.15 ± 9.64	44.21 ± 14.10	0.393
Donor age (years)	40.17 ± 7.81	44.46 ± 9.37	44.36 ± 7.87	0.349
Positive pretransplant PRA (*n*)	0	0	0	—
Previous transplant	0	0	0	—
Previous rejection, *n* (%)	0	0	0	—
Cold ischemic time (h)	8.22 ± 1.06	7.77 ± 1.17	8.00 ± 0.88	0.471
Warm ischemic time (min)	6.61 ± 1.65	6.31 ± 1.32	6.71 ± 1.27	0.728
Induction with IL-2R antibody, *n* (%)	18 (100)	13 (100)	14 (100)	—
Baseline immunosuppressants				0.224
MMF + Tac + Pred	13	10	8	
MMF + CsA + Pred	5	3	3	
Others	0	0	3	
Time of biopsy (day)	8 (5–20)	15 (11–25)	14 (12–15)	0.14

Abbreviations: ABMR: antibody-mediated rejection; TCMR: T cell-mediated rejection; SG: stable graft; PRA: panel-reactive antibody; IL: interleukin; MMF: mycophenolate mofetil; Pred: prednisolone; Tac: tacrolimus; CsA: cyclosporine A.

**Table 2:** Histological characteristics in different groups.

	ABMR (*n* = 18)	TCMR (*n* = 13)	SG (*n* = 14)	*Post hoc *		
				*P*_12_	*P*_13_	*P*_23_
Histological lesions						
PTC inflammation, *n* (%)	18 (100)	0	0	—	—	—
Glomerulitis, *n* (%)	18 (100)	0	0	—	—	—
Tubulitis, *n* (%)	18 (100)	13 (100)	6 (42.9)	—	<0.001	<0.001
Intimal arteritis, *n* (%)	16 (88.9)	5 (38.5)	0	<0.001	<0.001	0.005
Intraglomerular infiltrating cells count (cells/mm^2^)	5.90 (1.76–9.30)	0.30 (0.00–2.03)	0.00 (0.00–0.60)	0.001	<0.001	0.518
Interstitial						
CD4 (cells/mm^2^)	322 (165–426)	276 (181–372)	98 (69–147)	0.477	<0.001	<0.001
CD8 (cells/mm^2^)	266 (171–352)	204 (160–424)	96 (45–129)	0.863	<0.001	<0.001
CD4/CD8	1.16 (0.78–1.54)	1.15 (0.97–1.23)	1.18 (0.95–1.33)	0.766	0.849	0.655
CD68 (cells/mm^2^)	624 (398–924)	476 (202–614)	94 (50–194)	0.110	<0.001	<0.001
HLA-DR	0.36 (0.12–0.67)	0.20 (0.00–0.67)	0.00 (0.00–0.06)	0.365	0.004	0.049

Abbreviations: ABMR: antibody-mediated rejection; TCMR: T cell-mediated rejection; SG: stable graft; HLA: human lymphocyte antigen; PTC: peritubular capillary.

*P*_12_ means *P* value for ABMR group and TCMR group; *P*_13_ means *P* value for ABMR group and SG group; *P*_23_ means *P* value for TCMR group and SG group.

**Table 3:** BANFF scoring in different groups.

	ABMR (*n* = 18)	TCMR (*n* = 13)	SG (*n* = 14)	*Post hoc *		
				*P*_12_	*P*_13_	*P*_23_
PTC inflammation				<0.001	<0.001	—
0	0	13 (100%)	14 (100%)			
1	2 (11.1%)	0	0			
2	11 (61.1%)	0	0			
3	5 (27.8%)	0	0			
Glomerulitis				<0.001	<0.001	—
0	0	13 (100%)	14 (100%)			
1	1 (5.6%)	0	0			
2	8 (44.4%)	0	0			
3	9 (50.0%)	0	0			
Tubulitis				0.126	<0.001	<0.001
0	0	0	8 (57.1%)			
1	3 (16.7%)	5 (38.5%)	6 (42.9%)			
2	12 (66.7%)	7 (53.8%)	0			
3	3 (16.7%)	1 (7.7%)	0			
Intimal arteritis				<0.001	<0.001	0.020
0	2 (11.1%)	8 (61.5%)	14 (100%)			
1	6 (33.3%)	5 (38.5%)	0			
2	10 (55.6%)	0	0			
3	0	0	0			

Abbreviations: ABMR: antibody-mediated rejection; TCMR: T cell-mediated rejection; SG: stable graft; PTC: peritubular capillary.

*P*_12_ means *P* value for ABMR group and TCMR group; *P*_13_ means *P* value for ABMR group and SG group; *P*_23_ means *P* value for TCMR group and SG group.

**Table 4:** Capillary variation in different groups.

	ABMR (*n* = 18)	TCMR (*n* = 13)	SG (*n* = 14)	*Post hoc *		
				*P*_12_	*P*_13_	*P*_23_
Peritubular capillary						
Density (capillaries/field)	22.22 ± 2.51	27.23 ± 2.49	25.64 ± 1.82	<0.001	<0.001	0.105
Diameters (*μ*m)	2.34 ± 0.81	1.21 ± 0.49	1.09 ± 0.29	<0.001	<0.001	0.626
Intra-PTC area/field (%)	3.52 ± 1.76	2.30 ± 1.23	2.01 ± 0.56	0.026	0.006	0.608
Glomerulus						
Area (×10^4^ *μ*m^2^)	2.16 ± 0.36	2.29 ± 0.29	2.06 ± 0.44	0.274	0.512	0.097
Diameters of loops	165.50 ± 13.59	171.58 ± 11.18	162.14 ± 16.28	0.248	0.503	0.093

Abbreviations: ABMR: antibody-mediated rejection; TCMR: T cell-mediated rejection; SG: stable graft.

*P*_12_ means *P* value for ABMR group and TCMR group; *P*_13_ means *P* value for ABMR group and SG group; *P*_23_ means *P* value for TCMR group and SG group.

**Table 5:** Capillary variation between ABMR and TG.

	ABMR (*n* = 18)	TG (*n* = 32)	*P*
Peritubular capillary			
Density (capillaries/field)	22.22 ± 2.51	23.87 ± 3.92	0.115
Diameters (*μ*m)	2.34 ± 0.81	6.35 ± 2.16	<0.001
Intra-PTC area/field (%)	3.52 ± 1.76	9.53 ± 3.16	<0.001
Glomerulus			
Area (×10^4^ *μ*m^2^)	2.16 ± 0.36	3.48 ± 0.64	<0.001
Diameters of loops	165.50 ± 13.59	212.43 ± 25.20	<0.001

Abbreviations: ABMR: antibody-mediated rejection; TG: transplant glomerulopathy; PTC: peritubular capillary.

## Abbreviations

ABMR: Antibody-mediated rejection
TCMR: T cell-mediated rejection
TG: Transplant glomerulopathy
PTC: Peritubular capillary
SG: Stable graft
HLA: Human lymphocyte antigen.
